# Role of Nutraceuticals in Hypolipidemic Therapy

**DOI:** 10.3389/fcvm.2015.00022

**Published:** 2015-05-11

**Authors:** Carlo M. Barbagallo, Angelo Baldassare Cefalù, Davide Noto, Maurizio R. Averna

**Affiliations:** ^1^Biomedical Department of Internal Medicine and Specialistics (DIBIMIS), University of Palermo, Palermo, Italy

**Keywords:** nutraceuticals, lipids, LDL-cholesterol, hypolipidemic therapy, cardiovascular prevention

## Abstract

Nutraceuticals are food components or active ingredients present in foods and used in therapy. This article analyzes the characteristics of the molecules with a lipid-lowering effect. The different nutraceuticals may have different mechanisms of action: inhibition of cholesterol synthesis primarily through action on the enzyme HMG-CoA reductase (policosanol, polyphenols, garlic and, above all, red yeast rice), increase in LDL receptor activity (berberine), reduction of intestinal cholesterol absorption (garlic, plant sterols, probiotics), and also the ability to interfere with bile metabolism (probiotics, guggul). Based on the different mechanisms of action, some nutraceuticals are then able to enhance the action of statins. Nutraceuticals are often used without relevant evidence: mechanisms of action are not clearly confirmed; most of clinical data are derived from small, uncontrolled studies, and finally, except for fermented red rice, there are no clinical trials which may document the relationship between these interventions and the reduction of clinical events. Therefore, among all nutraceuticals, it is necessary to extrapolate those having a really documentable efficacy. However, these kinds of treatments are usually well-tolerated by patients. Overall, subjects with a middle or low cardiovascular risk are the best indication of nutraceuticals, but they may also be useful for patients experiencing side effects during classical therapies. Finally, in consideration of the additive effect of some nutraceuticals, a combination therapy with classical drugs may improve the achievement of clinical targets. Thus, nutraceuticals may be a helpful alternative in hypolipidemic treatment and, if properly used, might represent a valid strategy of cardiovascular prevention.

Nutraceutics is an area of pharmacology regarding food components or active ingredients that may be used as therapeutic agents. This includes a large number of compounds, such as an active ingredient, food supplements (i.e., supplements the normal diet), and functional foods (i.e., foods enriched with components with specific therapeutic or protective functions), as well as preparations based on medicinal herbs. Most compounds are vegetable originated, but there are also substances with animal origin (e.g., fish oil). Recent studies have shown promising results for these drugs in various pathological complications such as diabetes, atherosclerosis, cardiovascular diseases, cancer, and neurological disorders. These conditions involve many changes, including alterations redox state, and most of nutraceuticals have antioxidant activity with the ability to counteract this situation. Hence, nutraceuticals are considered as sources of health promotion (1), and they, nowadays, have received a considerable interest. A market research recently proposed that the worldwide nutraceuticals market is expanding and would reach US $250 billion by 2018 (1). Since nutraceuticals are generally considered like “foods,” their use do not strictly follow the same rules of classical drugs and have not patent protection. Thus, a large amount of preparations have been suggested to have a therapeutic effect and are rapidly available for patients. The process of market release for a drug is a very lengthy process, starting from the “*in vitro*” demonstration of the possible effects, followed by evaluation in animal models and then in humans (in healthy volunteers first and then in patients with a specific disease) analyzing effectiveness and tolerability of therapies (2). Following the approval and the market availability, there is also a strict monitoring of side effects. Large controlled clinical trials will finally validate clinical outcomes. In contrast, nutraceuticals are used in therapy without relevant evidence. They might be involved in a wide variety of biological processes, including activation of signal transduction pathways, antioxidant defenses, gene expression, cell proliferation, differentiation, and preservation of mitochondrial integrity, but the mechanistic actions are not always fully clear and sometimes they are not, or not particularly robust, for the theoretical basis of their effectiveness. Clinical efficacy often derives only from data produced by small-scale, uncontrolled studies. Thus, among all nutraceuticals, it is necessary to extrapolate those having a really documentable efficacy. Also, the “natural” origin does not protect from side effects in itself, since many classical drugs derive from plants and, in nature, there are also a number of toxic substances (such as derived from mushrooms); moreover, the insufficient clinical monitoring makes adverse events less predictable.

There are a variety of nutraceuticals with a potential lipid-lowering effect (Table 1), and therefore useful in the cardiovascular prevention (3). Nevertheless, in relation to the scarcity of experimental studies, these molecules do not always have solid scientific evidence with regard to both mechanisms of action and clinical efficacy (4). Data produced by small studies were often disavowed by larger and controlled studies or meta-analytic data. Some nutraceuticals are also able to enhance the action of the classic drugs (including statins), due to different mechanisms of action. Finally, except for the red yeast rice, there are no clinical trials which may document the relationship between any of these treatments and the reduction of clinical events, and this represents a great limit in their reasoned prescription (Table 2).

**Table 1:** **Mechanisms of action of different hypothetical nutraceuticals with recommended lipid-lowering effect**.

	Hepatic cholesterol synthesis			LDL uptake	Intestinal cholesterol absorption	Bile acids metabolism	
	HMG-CoA reductase	ACAT2	MTP	PCSK9		BSH	FXR
Policosanols	↓						
Poliphenols	↓	↓	↓				
Garlic	↓				↓		
Probiotis					↓	↑	
Plant sterols					↓		
Guggul							↓
Berberine				↓			
Red yeast rice	↓						

*HMG-CoA reductase, 3-hydroxy-3-methylglutaryl-coenzyme A reductase; ACAT2, acetyl-CoA acetyltransferase 2; MTP, microsomal transfer protein; PCSK9, proprotein convertase subtilisin/kexin type 9; BSH, bile salt hydrolase; FXR, farnesoid X receptor*.

**Table 2:** **Summary of clinical data of different nutraceuticals with recommended lipid-lowering effect**.

	Lipid-lowering effects hypothesized in clinical studies	Effectiveness confirmed in controlled studies	Clinical trials
Policosanols	LDL-C 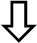 about 25%	No	No
Poliphenols	LDL-C 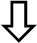 up to 30%	No	No
	Triglycerides 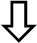 about 40%	
Garlic	LDL-C 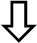 9–12%	No	No
Probiotis	LDL-C 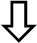 up to 40%	No	No
Plant sterols	LDL-C 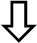 about 25%	No	No
Guggul	LDL-C 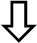 5–15%	Yes	No
Berberine	LDL-C 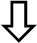 25% Triglycerides 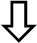 35%	Yes	No
Red yeast rice	LDL-C 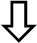 20–30%	Yes	Yes

Here, we review the major nutraceuticals with lipid-lowering effect: this should also include omega-3 polyunsaturated fatty acids (fish oil), but the scientific history of this drug followed pathways different than that of other nutraceuticals, and therefore will not be considered in this review.

## Policosanols

These are a mixture of natural long chain aliphatic alcohols obtained from a wide variety of plants. It has been suggested that policosanols might inhibit the activity of HMG-CoA reductase, but this is not definitively confirmed (5). In the early 90s, a number of clinical studies suggested a lipid-lowering effect of policosanols in different types of patients (healthy volunteers, hypercholesterolemics, diabetics, or postmenopausal women), with reduction in LDL-cholesterol similar to that of statins (about 25%), and a 10% increase of HDL-C (6). Some reports suggested benefits even on clinical outcomes, including coronary ischemia or claudication; these treatments appeared also very well tolerated by patients (7, 8). Nevertheless, studies were of limited samples but especially on a limited number of clinical centers, often in Cuba, with reduction in LDL-cholesterol similar to that of statins (about 25%), and a 10% increase of HDL-C (9, 10).

## Polyphenols

This is a very large family of substances available in the plant world. The main feature is the presence of multiple phenolic groups having a potent anti-oxidant effect; for this reason, polyphenols present in some foods typical of the Mediterranean diet (olive oil, red wine, fruits, vegetables) are considered to account for the protective effect of this nutritional model; drug preparations, maybe for a different bioavailability, do not seem to have the same clinical effect (11). It has also been postulated that polyphenols are able to inhibit HMG-CoA reductase, as well as ACAT2 and MTP, justifying a hypocholesterolemic effect (12). Nevertheless, even if in an open study, Mollace and colleagues have suggested the possibility of a great reduction of LDL-C (>30%) and also triglycerides levels (>40%) with polyphenols extracted from bergamot (13), a controlled study failed to demonstrate any effect in subjects treated with two different polyphenols (hesperidin and naringin) compared to a group on placebo (14). Thus, the hypolipidemic effect of polyphenols still remains an open question.

## Garlic

Allicin, a substance contained in the bulb of garlic, seems to be able to reduce both synthesis (perhaps through the inactivation of HMG-CoA reductase) and intestinal absorption of cholesterol (15), and therefore to have lipid-lowering properties, with reductions of total cholesterol reported between 9 and 12% (16). These data have recently been substantially refuted by Gardner and colleagues: these authors, in a randomized placebo-controlled trial, have not documented any cholesterol-lowering effect with different formulations of garlic (17). Overall, garlic, even without a significant lipid-lowering effect, could have other protective effects on the cardiovascular system for its ability to reduce blood pressure and platelet aggregation (18), but this needs to be better investigated in large controlled trials.

## Probiotics

They have received a lot of attention due to the potential benefits that they seem to have in different fields. Regarding lipid metabolism, probiotics could lower cholesterol absorption through direct cellular effects or mediated by bile metabolism (19). Several studies on different patients have documented significant reductions in total cholesterol, up to 40% (20–22). However, exact mechanisms of action were not identified, and those proposed (such as the inhibition of intestinal absorption of cholesterol) are usually dependent on bacterial strains and methods of execution of experiments, often very different from the “*in vivo*” conditions. More recently, it has been postulated a role of expression of the gene of bile salt hydrolase activity in the lactobacilli strains to explain the cholesterol-lowering action, even if this hypothesis itself does not seem completely convincing (23). A number of confounding factors, which also include local conditions or functional anatomy, make complex the question and, at the present time, additional data are certainly necessary to give a definitive answer.

## Guggul

This is a resin extracted from the bark of Commiphora mukul, a small thorny tree, also known as the tree of myrrh, used medically in India for hundreds of years (24). The active components, guggulsterone E and Z, have been demonstrated to have an antagonistic action of FXR, a nuclear receptor involved in the bile metabolism. Based on this data, published in *Science* in 2002 (25), and given the close relationship between cholesterol and bile metabolisms, it has been also proposed a role in modulating plasma lipid levels. Different studies, mostly uncontrolled trials, were conducted in India and showed a massive efficacy of guggul in LDL-cholesterol levels reductions with no effect on triglyceride or HDL-cholesterol concentrations (26). More recently, a randomized placebo-controlled study in healthy subjects with hyperlipidemia demonstrated no effect on plasma lipids of guggul, which also caused allergic reactions in some individuals as well (27), clarifying that guggul not only does not seem to have a real lipid-lowering effect but it could also be dangerous in predisposed individuals.

## Plant Sterols

Plant sterols decrease intestinal absorption of cholesterol through the reduction of the content of cholesterol within the micelles and a consequent lower proportion of absorbable cholesterol (28). In addition, some studies suggest that phytosterols are able to compete with cholesterol in the carrier of the intracellular incorporation (NPC1L1), and also increase the activity of transmembrane proteins responsible for the excretion of cholesterol (ABCA1) and plant sterols (ABCG5 and ABCG8) in the intestine and liver, with the net effect of increasing the release of both sterols into the intestinal lumen by enterocytes and in the bile ducts in the liver (29, 30). The lower intestinal absorption of cholesterol induced by plant sterols decreases cholesterol pool of liver, which responds by increasing the expression of LDL receptors, finally resulting in higher uptake of plasma LDL and therefore in a net hypocholesterolemic effect.

For many years, there was a great interest in phytosterols, which led to the development of a rich scientific literature. Phytosterols have been added and investigated in different food carriers. Initially, they were tested in margarines but other carriers in which it is possible to add plant sterols were subsequently identified: oils, salad dressings, meat products, low-calorie beverages, cereals, and finally drinks based on fermented milk (31–33). Clinical studies with beverages based on fermented milk enriched with phytosterols have shown that the effectiveness of phytosterols in reducing cholesterol is almost equivalent to that obtained with their introduction into margarine (34).

The cholesterol-lowering properties of phytosterols have been documented in a series of clinical trials in different categories of subjects: normolipidemic and hypercholesterolemic adults with and without cardiovascular disease, patients with type 2 diabetes, and children with familial hypercholesterolemia (35–39). In a multicenter Italian study, 1.6 g phytosterols/day, taken with a fermented milk, produced a reduction of LDL cholesterol from 166.2 ± 2.0 to 147.4 ± 2.8 mg/dL, and at the same time had an anti-oxidant effect, demonstrated by a significant reduction in the levels of isoprostane (40). Overall, clinical data suggest that the daily consumption of 1–3 g of plant sterols reduces LDL-cholesterol by 5–15%, with no significant effects on HDL cholesterol or triglycerides (41). Many clinical trials in patients with hypercholesterolemia treated with statins or fibrates are also available. The associations of plant sterols-fortified foods with these drugs determine an additive effect on the reduction in plasma levels of total cholesterol and LDL-cholesterol (41, 42). Some doubt, however, remains on safety. Beyond a possible interference with the absorption of fat-soluble vitamins, some observational studies have suggested an independent association between plasma levels of plant sterols and the risk of coronary heart disease. However, *in vivo* studies in animals fed with high doses of plant sterols seem to show a different effect, atherosclerosis, in terms of both, development of new plaques and size or lipid accumulation of lesions seem to improve (43). Recently, it has been suggested that the state of cholesterol “absorber” (as opposed to the state of “synthesizer”) may be the real atherogenic condition: thus, elevated plasma levels of phytosterols should indicate a hyperabsorptive pattern which might increase the risk (44). Longitudinal data in humans are not conclusive, and therefore this issue appears far from solved and the possibility of an increased of coronary risk, even if not likely, remains a problem to keep into account during this kind of treatment.

## Berberine

This substance, with a bitter taste and intense yellow color, is present in the bark, roots and stems, including underground (rhizome) of plants of the genus *Berberis*, such as barberry (*Berberis vulgaris* L.). For the antimicrobial and antisecretive properties, berberin is traditionally used in the treatment of infections (45). In recent years, most attentions have been on the metabolic properties of berberine. In 2004, Kong et al., have shown that berberine reduced plasma cholesterol by 29%, triglycerides by 35%, and LDL cholesterol by 25%, whereas did not modify HDL-cholesterol levels (46). Berberine increases the number of LDL receptors on the hepatic cell surface, similarly to statins. However, during statin therapy, the exposure of LDL-receptors on cell membranes follows the decrease of the endogenous cholesterol synthesis and the subsequent reduction of intracellular cholesterol pool, whereas the action of berberine seems linked to the ability to inhibit a protein (PCSK9) responsible for the partial degradation of LDL receptors in the liver. For these reasons, berberine may have synergistic effects with statins (47, 48). It is relevant to remember that inhibition of PCSK9 by monoclonal antibodies is a new lipid-lowering strategy and several clinical trials are currently in progress (49, 50). Synergy between berberine and statins has been demonstrated by both cell culture and experimental animals studies. In humans, Bertolini et al. documented additive effects of treatment with berberine/statins in patients with heterozygous familial hypercholesterolemia, even higher to those of ezetimibe/statins; a recent meta-analysis confirmed the cholesterol-lowering effect of berberine, indicating also the necessity of further data from randomized trials (51). Other studies have even highlighted hypoglycemic effects of berberine in patients with diabetes mellitus type 2 by increasing the expression of insulin receptors and the improving of insulin resistance (52). The action on PCSK9, the ability to operate at multiple levels and the absence of significant side effects, suggests that berberine represents one of the most interesting available nutraceutic drugs.

## Fermented Red Rice

The fermentation of red rice by a fungus (*Monascus purpureus*) produces a substance called monacolin K, which inhibits the synthesis of cholesterol (53). The monacolin K is also known as lovastatin, a statin available in the market worldwide. The red yeast rice also produces other monacolins that may enhance the inhibition of HMG-CoA reductase. In addition, recent data show that, compared to the classical lovastatin, monakolin K extracted from red yeast rice have even a higher bioavailability with a higher efficacy at the same dosage (54).

Red yeast rice represents an important drug of traditional Chinese medicine, and a considerable amount of experimental data have shown the efficacy and tolerability of red yeast rice. Heber et al. in a controlled study in 83 healthy hyperlipidemic subjects treated with 2.4 g/day of red yeast rice or placebo, observed large reductions in LDL-cholesterol (39 ± 19 mg/dL), significantly different from placebo (55). Studies in other populations, as summarized by Liu et al. in a meta-analysis of 93 randomized trials, led to similar results, demonstrating a significant effect on total and LDL-cholesterol levels but not on triglycerides or HDL-cholesterol concentrations (56). In a study that evaluated the efficacy of simvastatin (40 mg/day) vs. alternative treatments (including red yeast rice) for 12 weeks in dyslipidemic subjects with a history of statin myalgias, Becker et al. showed comparable reduction of LDL-C in the two groups. These authors assessed also tolerability, measured either as changes in biochemical parameters or pain severity, and, behind the cholesterol lowering efficacy, observed a good tolerability of red yeast rice (57). Red yeast rice was even similar to pravastatin in the reduction of LDL-C (27 vs. 30%), but it was associated with a lower rate of withdrawn from the treatment (58). Overall, red yeast rice seems to have a lower incidence of muscle disorders than statins. Myotoxicity, in the form of myopathy, myalgia, myositis, or rhabdomyolysis, is the most severe adverse effect of statins and the main cause of discontinuation. Statin therapy is associated with muscle problems in approximately 10–25% of patients treated in clinical practice (59). The exact pathophysiology of statin-induced myopathy is not fully known, and multiple mechanisms may contribute to that. One of these should be represented by a possible depletion of mevalonate metabolites (such as ubiquinone), and red yeast rice might be less efficient in blocking this metabolic cascade. Psychological components may also have a role, and the possibility to replace statins with natural drugs may have the consequent effect of removing this anxiety (60). Thus, red rice might be a real therapeutic alternative in patients intolerant to statins. Recently, in a group of hypercholesterolemic patients with a mild/moderate risk previously intolerant to statins or refusing classical pharmaceutical treatment, we examined in a double-blind, placebo-controlled, randomized study, the efficacy, safety, and tolerability of a product containing red yeast rice, but also the pulse wave velocity as expression of arterial stiffness and endothelial function. Patients received daily either a nutraceutical-combined pill, containing red yeast rice 200 mg (corresponding to monakoline 3 mg) or placebo for 6 weeks, and we observed, among subjects treated with red yeast rise, a significative reduction of total cholesterol and LDL-cholesterol (respectively by 10.4 and 12.2%), as well as of pulse wave velocity (by 6.5%); triglyceride and HDL-cholesterol levels did not change. Safety parameters did not change during the study and no patient reported muscle pain (61). Finally, in a 5-year long secondary prevention trial in 5000 Chinese subjects, Lu and colleagues demonstrated that an extract of red yeast rice, the Xuenzhikang (XZK), beside a 20% decrease of LDL-cholesterol, led to a significant reduction of coronary events in comparison with placebo (5.7 vs. 10.4%), with a reduction of relative and absolute risk of 45 and 4.7%, respectively, and together with high tolerability of treatment (62). To date, this unique trial of cardiovascular prevention with a nutracetical drug is available.

Recent European Guidelines of cardiovascular prevention include treatment with nutraceuticals for their clinical effects and tolerability (63). However, it should be emphasized that some categories of patients may particularly benefit from treatment with nutraceuticals. The best indication is subjects with a middle or lower cardiovascular risk in whom a modest reduction in LDL-C may be sufficient to reach LDL-C levels internationally recommended to reduce the risk (the so-called “target values”). In these patients, usually only on dietary treatment, therapy with nutraceuticals allows a better, rapid, and more stable achievement of aims. Also, subjects experiencing side effects with statins may be treated by nutraceuticals. In these subjects, a small but stable LDL-cholesterol reduction may be useful when statin therapy at standard doses is not feasible. Another potential use of those nutraceuticals having a mechanism of action different form statins, e.g., berberin or plant sterols, is the combination therapy with statins in order to either reduce statin dose or increase the hypolipidemic efficacy. The clinical use of these preparations may be also extended to other categories of patients. In those who are afraid of side effects of classical drugs or simply do not want to be considered a patient, the psychological effect of a “natural” treatment may be helpful to permit to treat these subjects and therefore to reduce their risk level. The cost issue raised by the treatment with nutraceuticals is still unresolved, since these therapies are usually more expensive than classical drugs and, in order for them to be efficient in reducing cardiovascular risk, should be lifelong.

In conclusion, nutraceuticals represent a valid alternative hypolipidemic treatment, and thus have a role in cardiovascular prevention strategies. Nutraceuticals have multiple physiological benefits and their use may be a valid alternative or complementary therapy to conventional treatment in many fields. A proper and reasoned use may help to prevent chronic diseases, increase life expectancy, support the structure or function of the body, delay the aging process, and help maintain overall good health (64). Due to the growing industrial and commercial interest in the future, it is desirable to have better regulations and improve the scientific agreement to warrant safe usage and clinical effectiveness (65).

## Conflict of Interest Statement

The authors declare that the research was conducted in the absence of any commercial or financial relationships that could be construed as a potential conflict of interest.
